# The importance of mammillary body efferents for recency memory: towards a better understanding of diencephalic amnesia

**DOI:** 10.1007/s00429-016-1330-x

**Published:** 2016-10-25

**Authors:** Andrew J. D. Nelson, Seralynne D. Vann

**Affiliations:** School of Psychology, Cardiff University, 70 Park Place, Cardiff, CF10 3AT UK

**Keywords:** Anterior thalamic nuclei, Diencephalic amnesia, Mammillothalamic tract, Prefrontal cortex, Rats, Recognition memory

## Abstract

Despite being historically one of the first brain regions linked to memory loss, there remains controversy over the core features of diencephalic amnesia as well as the critical site for amnesia to occur. The mammillary bodies and thalamus appear to be the primary locus of pathology in the cases of diencephalic amnesia, but the picture is complicated by the lack of patients with circumscribed damage. Impaired temporal memory is a consistent neuropsychological finding in Korsakoff syndrome patients, but again, it is unclear whether this deficit is attributable to pathology within the diencephalon or concomitant frontal lobe dysfunction. To address these issues, we used an animal model of diencephalic amnesia and examined the effect of mammillothalamic tract lesions on tests of recency memory. The mammillothalamic tract lesions severely disrupted recency judgements involving multiple items but left intact both recency and familiarity judgements for single items. Subsequently, we used disconnection procedures to assess whether this deficit reflects the indirect involvement of the prefrontal cortex. Crossed-lesion rats, with unilateral lesions of the mammillothalamic tract and medial prefrontal cortex in contralateral hemispheres, were unimpaired on the same recency tests. These results provide the first evidence for the selective importance of mammillary body efferents for recency memory. Moreover, this contribution to recency memory is independent of the prefrontal cortex. More broadly, these findings identify how specific diencephalic structures are vital for key elements of event memory.

## Introduction

The medial diencephalon was probably the first brain region to be linked to memory loss (Gudden 1896), but there still remains much uncertainty over the core features of diencephalic amnesia and how structures within the medial diencephalon support mnemonic functions. Evidence from Korsakoff’s syndrome and lacunar infarct patients indicates that the mammillary bodies and thalamus are the primary locus of pathology in diencephalic amnesia (Carlesimo et al. 2011; Harding et al. 2000; Mayes et al. 1988; Pitel et al. 2012; Van der Werf et al. 2003). However, the pathology is rarely circumscribed and, particularly in the case of Korsakoff’s syndrome, there are often concomitant changes to both white matter tracts and grey matter structures beyond the medial diencephalon, including frontal lobe dysfunction (Harper and Corbett 1990; Harper 2009; Langlais et al. 1996; Torvik et al. 1982). Consequently, it has proved difficult to attribute specific cognitive impairments to particular brain regions (Kopelman 2015).

Diencephalic amnesic patients are impaired on tests of recency memory that require individuals to make judgments about the temporal context in which an item was encountered (Hildebrandt et al. 2001; Huppert and Piercy 1976; Kopelman et al. 1997; Meudell et al. 1985; Parkin et al. 1990). Temporal or recency memory is classically associated with the frontal cortex; both patients (McAndrews and Milner 1991; Milner et al. 1991; Shimamura et al. 1990) and animals (Barker et al. 2007; Petrides 1991) with damage to frontal regions are impaired on tasks that tax this aspect of memory. There are various possible explanations of impaired recency memory in diencephalic amnesia: it may be due to co-occurring frontal lobe dysfunction (Mayes et al. 1985; Shimamura et al. 1990; Squire 1982), it could reflect the disconnection between frontal cortex and frontal cortex-associated thalamic nuclei, such as the mediodorsal thalamus (Cross et al. 2012; Schnider et al. 1996), or alternatively, it might be a core feature of diencephalic amnesia (Hunkin and Parkin 1993; Kopelman 1989; Kopelman et al. 1997). Given the lack of patients with circumscribed diencephalic pathology, less equivocal evidence can only be obtained from animal models involving restricted damage within discrete regions of the medial diencephalon. In this respect, targeting the mammillothalamic tract (MTT) is particularly appealing, because all neurons in the mammillary bodies are thought to project to the anterior thalamus via this tract (Vann et al. 2007). MTT transection, therefore, allows a direct assessment of mammillary body contributions to medial diencephalic function.

To address these issues, we first tested rats with discrete lesions to the MTT on a variant of the recency tasks used with Korsakoff patients (Hunkin et al. 2015). This task makes use of rats’ inherent preference for relative novelty (Hannesson et al. 2004), to discriminate between multiple objects presented at different time points and makes it possible to test both “within-list” and “between-list” memory. Performance on this task (between-block recency) is sensitive to both hippocampal and anterior thalamic damage (Albasser et al. 2012; Dumont and Aggleton 2013). Control experiments assessed both recency and familiarity judgements for single items. Second, we examined whether any impairments seen in MTT lesion rats could reflect frontal involvement. Bilateral medial prefrontal damage is known to disrupt simple between-block recency discriminations (Barker et al. 2007; Hannesson et al. 2004; Nelson et al. 2011). The medial mammillary bodies are directly innervated by medial prefrontal cortex (Allen and Hopkins 1989). The medial mammillary bodies can also indirectly influence the frontal cortex via their connections with the anteromedial thalamic nucleus (Hayakawa and Zyo 1989; Wright et al. 2013). In turn, the anteromedial thalamic nucleus projects unilaterally to the medial prefrontal cortex (de Lima et al. 2016; Hoover and Vertes 2007; van Groen et al. 1999). Moreover, MTT lesions in rats can disrupt markers of neuronal activity (as measured by the immediate-early gene *c*-*fos*) in prelimbic cortex (Vann and Albasser 2009; Vann 2013). As such, any effects of bilateral MTT lesions on recency discriminations could, in part, be mediated by disruption to information flow within prefrontal-mammillary body-anteromedial thalamic-prefrontal pathways or by distal effects of MTT lesions on prefrontal functioning. Disconnection procedures can be used to rule out these explanations. Accordingly, we tested crossed-lesion rats, with unilateral lesions of the MTT and medial prefrontal cortex in contralateral hemispheres, on the same recency memory task.

## Materials and methods

### Subjects and surgery

In Experiment 1, subjects were 28 male Lister Hooded rats (Harlan, Bicester, UK) weighing between 234 and 303 g at the time of surgery. Experiment 2 involved an additional 28 male Lister Hooded rats (Harlan, Bicester, UK) weighing between 276 and 384 g at the time of surgery. Animals were housed in pairs under diurnal light conditions (14 h light/10 h dark) and testing was carried out during the light phase. Animals were given free access to water and a large cardboard tube and wooden chew-stick were available in the home-cage throughout. All experiments were carried out in accordance with UK Animals (Scientific Procedures) Act, 1986 and EU directive 2010/63/EU.

Surgery was performed under an isoflurane-oxygen mixture (2–2.5% isoflurane). Once anaesthetised, the animals were placed in a stereotaxic head holder (David Kopf Instruments, Tujunga, CA), with the nose-bar at −3.3 (flat skull), and a longitudinal incision was made in the scalp, which was retracted to expose the skull. The skull was drilled at the point of the lesion. In Experiment 1, rats received bilateral mammillothalamic tract lesions (MTTx; *n* = 15) made by radiofrequency using a thermocouple radiofrequency electrode (0.7 mm active tip length, 0.25 mm diameter; Diros Technology Inc., Toronto, Canada). The electrode was lowered vertically and the tip temperature raised to 70 °C for 33 s using an OWL Universal RF System URF-3AP lesion maker (Diros Technology Inc., Toronto, Canada). The stereotaxic co-ordinates for the lesions were: anterior–posterior (AP), −2.5; medio-lateral (ML), ±0.9 (both relative to bregma); and the depth (DV), from top of cortex, was −6.9 mm. The surgical control rats (Sham; *n* = 13) underwent the same procedures except the probe was lowered to +1.0 mm above the lesion site and the temperature of the probe was not raised.

In Experiment 2, 16 rats received crossed mammillothalamic tract—medial prefrontal cortex lesions (i.e., a unilateral mammillothalamic tract (MTT) lesion in one hemisphere and a medial prefrontal cortex (mPFC) lesion in the contralateral hemisphere). Half of the animals received an MTT lesion in the left hemisphere and an mPFC lesion in the right hemisphere, while for the remaining eight animals, this order was reversed. The intended locus of the unilateral mPFC lesion was selected on the basis of the known outputs from the mPFC to the mammillary bodies and inputs to the mPFC from the anteromedial thalamic nucleus (Allen and Hopkins 1989; Hoover and Vertes 2007; van Groen et al. 1999) as well as evidence from the previous lesion studies implicating the mPFC in recency memory (Barker et al. 2007; Devito and Eichenbaum 2011; Hannesson et al. 2004; Hasselmo and Eichenbaum 2005; Nelson et al. 2011). The MTT lesions were conducted in the same manner as for Experiment 1 except that the lesion was only made in one hemisphere and the tip temperature of the probe was raised to 70 °C for 35 s. Following a craniotomy at the point of the lesion site, the unilateral mPFC lesions were made by injecting three sites with 0.28 μl of 0.09 M *N*-methyl-d-aspartic acid (NMDA; Sigma). Infusions were made with a 1 μl Hamilton syringe (Bonaduz, Switzerland) at an infusion rate of 0.1 μl/min. The AP and ML co-ordinates were measured (in mm) with respect to bregma, and the DV co-ordinates (in mm) with respect to the surface of the cortex. The stereotaxic co-ordinates of the three injection sites were as follows: (1) AP = +3.8; ML = ±0.7; DV = −3.8; (2) AP = +3.2; ML = ±0.7; DV = −3.6; and (3) 3. AP = +2.5; ML = ±0.7; DV = −3.4. The needle was left in situ for 5 min after each infusion. The 12 surgical controls underwent a unilateral “sham” MTT surgery as described for Experiment 1 and a “sham” unilateral mPFC lesion which was conducted in the same manner as the lesion except that no infusions of NDMA were made.

On completion of surgery, the skin was sutured and antibiotic powder (Clindamycin Hydrochloride, Pharmacia, Sandwich, UK) was applied topically to the wound-site. Animals also received subcutaneous injections of 5 ml glucose saline and Metacam (0.06 ml, s.c.; 5 mg/ml Meloxicam, Boehringer Ingelheim, Rhein, Germany) provided post-operative analgesia. All animals recovered well following surgery.

After a minimum of 2-week post-operative recovery, rats were placed on a food restricted diet where they were still able to gain weight; their weights did not fall below 85% of their equivalent free feeding weight.

### Behavioural testing

#### Experiment 1a and 2: multi-item recency judgments

These experiments made use of rats’ spontaneous preference for less recently presented (i.e., more novel) objects as a measure for recency memory and involved both between- and within-list discriminations (Dumont and Aggleton 2013). Rats were required to discriminate, on the basis of relative recency, between pairs of objects that had either been presented in separate temporal blocks (between-block) or within the same continuous block of trials but at different time points (within-block).

Rats underwent each recency test (between- and within-block recency) twice. The order of testing was counterbalanced across animals, such that half of the animals were tested first on between-block recency, then on within-block recency followed by the second test of between-block recency and finally the second within-block recency test. For the other half of the animals, this order was reversed. At least 7 days separated each test.

##### Apparatus

**Fig. 1:**
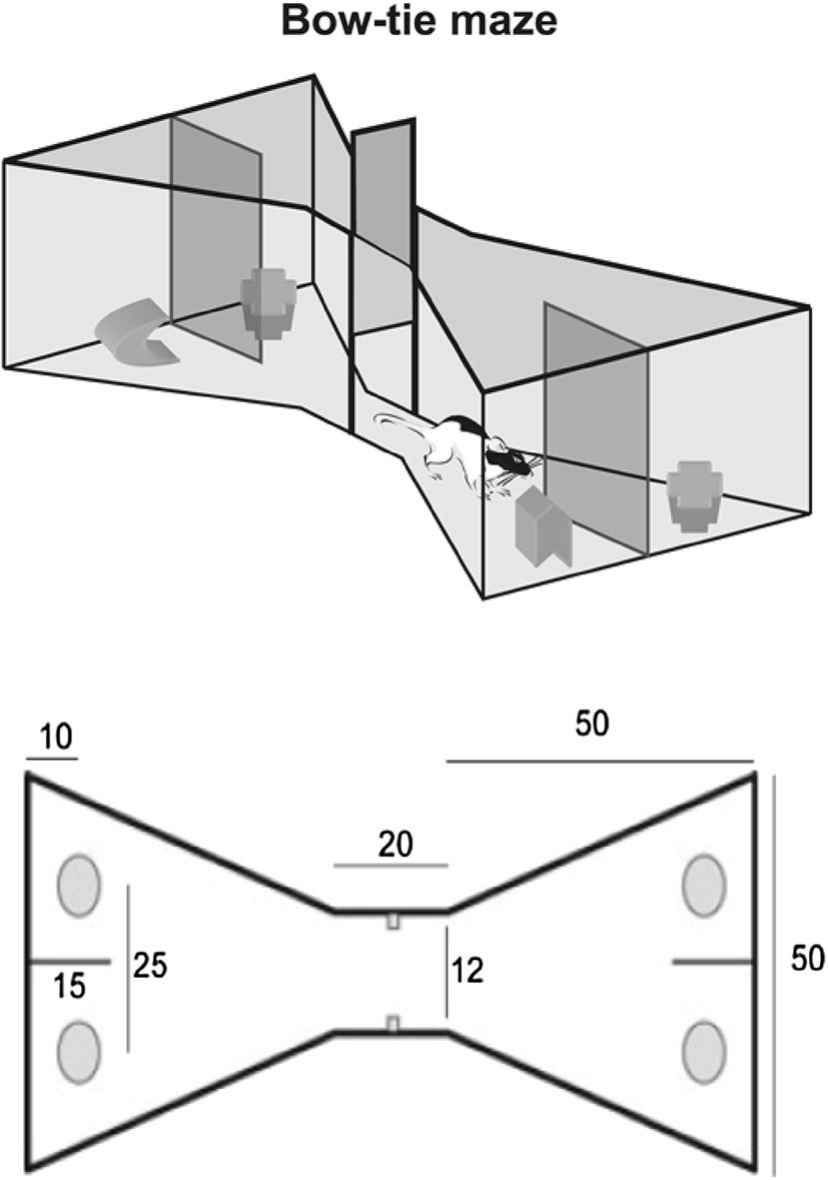
*Upper* panel is a graphic of the test apparatus used for testing object recognition and object recency memory. A sliding door in the centre divides the maze into two halves, so that objects can be placed over the food wells in one half, while the animal is completing the task in the other half. *Lower* panel is a schematic of the bow-tie maze, with dimensions in centimetres

Testing occurred in a maze with the shape of a bow tie (120 cm long, 50 cm wide, and 50 cm high) made of aluminium (Fig. 1; Albasser et al. 2010). Each end of the maze consisted of a triangular area, and these areas were joined together at their apices by a corridor (12 cm wide). In the centre of the corridor, an opaque sliding door could be lowered or raised by the experimenter to allow passage from one end of the maze to the other. At the far wall of each of the triangles, there were two food wells (3.5 cm in diameter and 2 cm deep), separated by a short, opaque, wall extending 15 cm from the middle of the end wall. The two food wells were 25 cm apart. Objects were placed above these two food wells during the experiment.

Triplicate sets of identical objects that differed in size, shape, colour, and texture were used. A mixture of plastic, glass, ceramic, and wooden objects was used. Objects had to be large enough to cover one food well but also light enough for the rats to displace. The height of the objects ranged between 2 and 15 cm and the width ranged between 4 and 10 cm. The presentation of objects was counterbalanced, so that half the rats experienced the list of objects in one order (e.g., A–K), whereas the other half of rats experienced the list in the reverse order (e.g., K–A). The positioning of the objects within the maze (over either left or right food well) was also counterbalanced. Different sets of objects were used for each test, so that each test contained unique items.

##### Habituation and pre-training

Habituation lasted 7 days during which time the rats learnt to run from one end of the maze to the other and displace objects covering the food wells to obtain a sucrose reward pellet. Initially (day 1), pairs of rats were allowed to explore the maze for 20 min and collect sucrose pellets that had been scattered across the floor and food wells. One the next day, individual rats were trained (10 min) to run back and forth for rewards that were now located in the food wells. On day 3, the sliding door that restricted movement from one compartment to the other was introduced. On day 4, the rats learnt to push objects to obtain the sucrose pellets by placing four identical wooden blocks that partially, and subsequently, fully occluded the food wells. For the remaining three sessions, different pairs of objects were introduced. None of the objects used during pre-training was subsequently used during testing.

##### Between-block recency

**Fig. 2:**
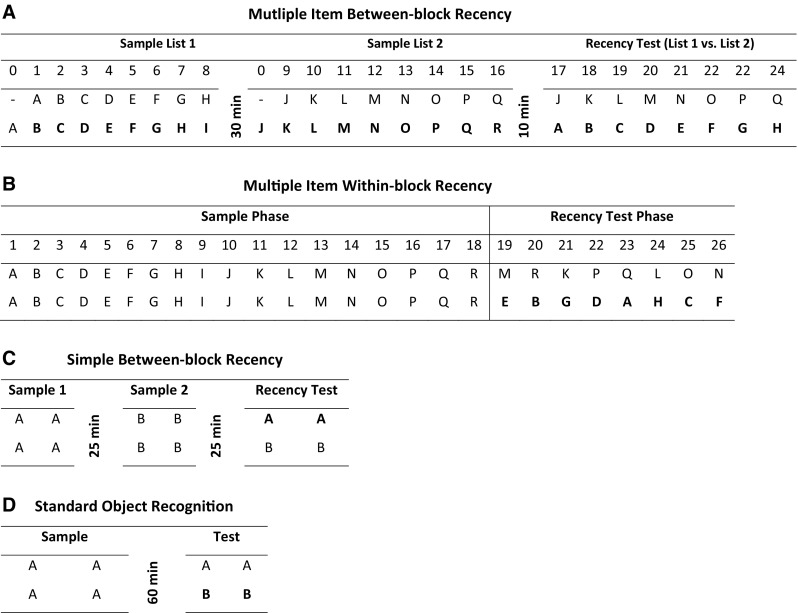
Order of object presentation in the multi-item between-block (**a**) and within-block (**b**) recency tests. Order of object presentation in the simple between-block (**c**) recency test and standard object recognition test (**d**). Items in bold refer to either novel or least recently explored objects, i.e., objects for which rats should show a preference

Each session consisted of three phases: two sample phases followed by a test phase (see Fig. 2a). Thus, rats were presented with lists of objects in two distinct, temporal blocks; at test, rats were required to discriminate between objects that had been presented in different temporal blocks. Each sample phase involved multiple trials of standard object recognition (Fig. 2a).

During Sample 1, rats received eight trials of standard object recognition (plus a trial “0” in which the first object was encountered). At the beginning of the session, the rat was placed in one end of the maze that contained an object (Object A1) covering one food well and a wood block covering the other well. The rat was allowed to retrieve the food rewards and explore both objects in a trial lasting 1 min. The sliding door was then raised allowing access to the second compartment. Once the rat ran to the opposite side of the maze the sliding door was lowered, the rat could now explore a novel item (Object B1) and a familiar item (Object A2, a duplicate of Object A1 from Trial 0). Both the novel and familiar objects covered wells that contained a reward, so every object on every trial was rewarded. After a minute, the sliding door was raised again, and the rat ran back to the first compartment of the maze (Trial 2) where Object C (C1; novel) and a duplicate of Object B (B2; familiar) were presented. After 1 min, the sliding door was raised again (Trial 3), and the rat ran back into the second compartment to explore a copy of Object C (C2; familiar) and new Object D (D1; novel). This process continued with different objects until 8 trials had been completed, i.e., objects A–I. Sample 2 followed after a 30 min delay and consisted of eight more trials of standard object recognition involving the new objects J–R (see Fig. 2a).

After a further 10 min delay, recency judgements were assessed by presenting rats in each trial with pairs of objects with one object from Sample 1 (less recent) and one from Sample 2 (more recent). The rat was returned to one end of the maze and copies of objects from each of the sample phases (e.g., Object A3 and Object J3) were presented. The rat had 1 min to explore the objects and retrieve the pellets from under both objects. The sliding door was then raised, and the rat ran to the second compartment where further copies of objects from each sample phase (e.g., B2 and K3) were presented. This procedure continued until all eight trials had been completed.

##### Within-block recency

Each session involved an 18-trial sample phase and an 8-trial recency test phase both of which occurred within a continuous block of trials.

The sample phase began when the rat was placed in one end of the maze. The rat was allowed 1 min to push aside and explore two identical objects (A1, A2) that each covered a food well (Fig. 2b). The sliding door was then opened, allowing the rat to run across to the second compartment where two copies of a novel object were present (B1, B2). The rat again had 1 min to explore these objects and obtain the sucrose pellets. Once this trial was completed, the sliding door was again opened and the rat ran back to the first compartment where two copies of novel another novel object (C1, C2) were presented. This process continued until the rats had encountered all 18 pairs of objects (i.e., A–R). Following the trial 18, the sliding door was raised allowing the rat to change compartments. The recency phase began immediately, so the rat was not removed from the apparatus or was the rat handled between two phases.

For each trial of the test phase, the rat could explore two objects that had been presented at different time points in the sample phase. As before, every object covered a food reward. For example, Trial 1 of the recency (test) phase consisted of copies of object E (E3) and object M (M3). After a minute, the rat was allowed to run to the other side of the maze to find copies of object B (B3) and object R (R3). The number of interleaving items between the two objects was set at 3, 7, 11, or 15. Trials with different numbers of interleaving items were intermixed.

#### Experiment 1b: single-item recency judgments

To reduce the proactive interference that arises from being presented with multiple different objects within a relatively short timeframe, this experiment assessed rats’ ability to discriminate between single items that had been presented in separate temporal blocks. Each rat was tested twice, with a minimum 7-day interval between each test. Different sets of objects were used in each test, and none of the objects had been encountered previously.

##### Apparatus

Testing occurred in the same bow-tie maze as described previously. Two sets of four identical objects were used. At test, two duplicates from each set were used. As previously, junk objects that differed in size, shape, colour, and texture were used.

##### Procedure

Each session consisted of three phases: two sample phases followed by a test phase (see Fig. 2c). This experiment did not involve multiple continuous trials, and consequently, no reward pellets were placed under the objects, so that the animals could not displace them, the objects were larger than those in Experiment 1a/b. The order of presentation, i.e., whether the object was presented in Sample 1 or 2 was counterbalanced across animals. In Sample 1, rats were placed in the arena and were able to move freely around the entire maze and to explore four identical objects. After 5-min exploration time, the rats were removed from the arena and were returned to a holding room for 25 min. After this delay, the rats were returned to the maze and could explore a new set of four identical objects for 5 min. Following a further 25 min delay, the test phase occurred during which rats were able to explore two pairs of objects (one pair from each sample phase—see Fig. 2c). Each end of the maze contained one replica object from each sample phase. The test phase lasted 3 min.

#### Experiment 1c: standard object recognition

This experiment examined standard object recognition memory, i.e., the ability to discriminate between items on the basis of relative familiarity. MTT lesions do not disrupt this ability with relatively short retention delays, e.g., 10 min (Nelson and Vann 2014). To match the maximum delay between object presentation in the recency tests (Experiments 1a/b), this experiment examined animals’ ability to discriminate a novel from a familiar object after a 60 min delay.

##### Apparatus

Testing occurred in the same bow-tie maze as described previously. Two sets of four identical objects were used. At test, two duplicates from each set were used. As previously, junk objects that differed in size, shape, colour, and texture were used.

##### Procedure

Each session consisted of two phases: a sample phase followed by a test phase (see Fig. 2d). This experiment did not involve multiple continuous trials, and consequently, no reward pellets were placed under the objects. The set of objects that served as familiar or novel was counterbalanced across animals. In Sample 1, rats were placed in the arena and were able to move freely around the entire maze and to explore four identical objects. After 5-min exploration time, the rats were removed from the arena and returned to a holding room. After a 60-min delay, the test phase occurred during which rats were able to explore a pair of objects previously encountered in the sample phase (familiar objects) and two identical novel objects (Fig. 2d). Each end of the maze contained one novel and one familiar object. The test phase lasted 3 min.

### Histology

At the end of the behavioural experiments, the rats were deeply anaesthetised with sodium pentobarbital (60 mg/kg, Euthatal, Rhone Merieux, UK) and transcardially perfused with 0.1-M phosphate buffer saline (PBS) followed by 4% paraformaldehyde in 0.1 M PBS (PFA). The brains were removed and post-fixed in PFA for 4 h and then transferred to 25% sucrose overnight at room temperature with rotation. Sections were cut at 40 μm on a freezing microtome in the coronal plane.

A one-in-four series of sections was mounted onto gelatin-coated slides and stained with cresyl violet, a Nissl stain, for histological assessment. A second series was collected to process for the visualization of calbindin (Arai et al. 1994; Rogers and Résibois 1992). The dense fibrous calbindin stain within the anteroventral thalami nucleus has been attributed to MTT input (Rogers and Résibois 1992); this stain can, therefore, provide a further measure of the completeness of the MTT lesions. The tissue was treated with a blocking buffer containing 3–5% normal horse serum (S-2000, Vector Laboratories, UK) in 0.1 M PBS and agitated on a stirrer for between 30 min and 2 h. Sections were subsequently incubated in primary antibody solution (Swant, Switzerland) (1:10,000 dilutions in 0.2% Triton-X-100 in PBS containing 1% normal horse serum), for 24 h at room temperature. The tissue underwent further washes in 0.1 M PBS and, to complete the reaction, the tissue was incubated in a secondary antibody solution (Dylight-594; horse, anti-mouse; 1:200 dilution in 0.2% Triton-X-100 in 0.1 M PBS containing 1% normal horse serum) overnight on a shaker table at room temperature. Following an additional series of washes in 0.1 PBS, the tissue sections were mounted on gelatin-subbed slides, allowed to dry for 1–2 days in the dark, and coverslipped using DPX mounting medium (Lamb, UK).

### Data analysis

Exploration of an object was defined as directing the nose at a distance of <1 cm to the item and/or touching it with the nose or the paws (including pushing). Sitting on or turning around the item was not included. If the rats spent time chewing, carrying the items in their mouths, and freezing near or above the items (at a distance of <1 cm), these behaviours were also excluded. The videos were scored blind to lesion group assignment.

A discrimination score (D1) and a ratio (D2) were calculated (Ennaceur and Delacour 1988). The recognition score D1 was calculated by subtracting the time spent exploring the older item from the time spent exploring the recent item. When there were multiple trials (between- and within-block recency), the D1 index was summed across trials (cumulative D1). The D2 ratio takes the differential exploration time for the pair of objects (i.e., the D1 score) and then divides it by the total time spent exploring both items. The D2 ratio yields a ratio between −1 and +1, where a positive score indicates a preference for the least recent (older) item. For the tests of between- and within-block recency, which involved multiple trials, the D2 ratio was updated after every trial using the summed (updated D2) data (note that the final updated D2 score is, therefore, not equivalent to the mean of each D2 score for every trial).

The D1 score and D2 ratio were also calculated for the standard object recognition trials (Experiment 1a between-block recency sample phase, Experiment 1c). The time spent exploring the novel item was subtracted from the time spent exploring the familiar item (i.e., time novel–time familiar). For the D2 ratio, a positive score indicates a preference for the novel item.

Group differences were examined with between subject ANOVAs. To verify whether animals’ performance was above chance (i.e., zero), the D1 scores and D2 ratios were compared against zero, using a one-sample *t* test. The alpha level was set at *p* < 0.05.

## Results

### Histological analysis of the lesions

**Fig. 3:**
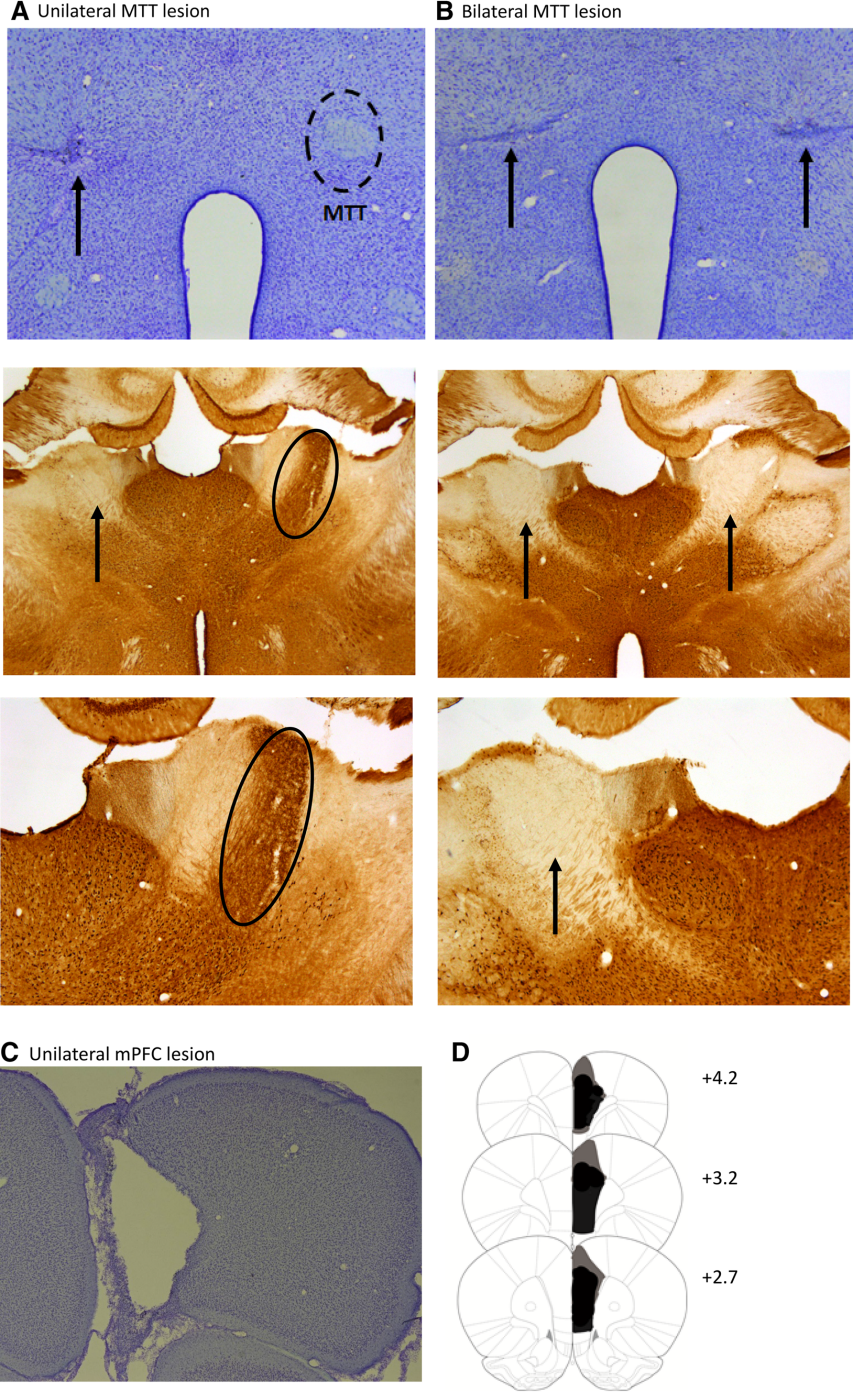
Location and histological verification of mammillothalamic tract (MTT) and medial prefrontal cortex (mPFC) lesions. Photomicrograph of a coronal section immunostained for Nissl (*top* panel) and for calbindin in the anterior thalamus (*middle* and *bottom* panels) showing a unilateral MTT lesion (**a**) and a bilateral MTT lesion (**b**). Note the marked loss of calbindin stain in the anteroventral nucleus in the MTT lesion hemispheres. **c** Photomicrograph of a coronal section stained for Nissl showing a unilateral mPFC lesion. **d** Coronal reconstructions showing cases with the minimal (*black*) extent and the maximal (*black* and *grey* areas) extent of the unilateral mPFC lesions. The* numbers* in (**d**) indicate the distance (in millimeters) from bregma (adapted from Paxinos and Watson 2005)

The MTT lesions were quantified on the basis of Nissl-stained sections and the absence of calbindin staining in the anteroventral thalamic nucleus (Fig. 3a, b). In Experiment 1, 5 of the 15 lesion animals did not have complete bilateral MTT lesions and were consequently removed from all analyses. In Experiment 2, there was evidence of sparing in 7 cases and so these animals were also removed from all analyses. All remaining cases involved discrete bilateral (Experiment 1) or unilateral (Experiment 2) lesions of the MTT, which were sufficiently anterior, so there was no direct damage to the supramammillary nuclei, the mammillary bodies, or the mammillotegmental tract. Similarly, the lesions did not encroach on the postcommissural portion of the fornix (Fig. 3a, b). In Experiment 2, the nine unilateral MTT lesions animals also received a unilateral mPFC lesion in the contralateral hemisphere to the MTT lesion site. The animals exhibited substantial unilateral cell loss within the mPFC. The entire infralimbic cortex was atrophied, with damage also extending into the dorsal peduncular cortex. The prelimbic cortex as well as the rostral anterior cingulate cortex were also absent, except in one case where there was substantial sparing in the dorsal aspect. This animal was, therefore, excluded, leaving 8 cases with crossed MTT-mPFC lesions.

### Behavioural results

#### Experiment 1a: The effect of MTT lesions on multi-item recency judgments

##### Between-block recency

**Fig. 4:**
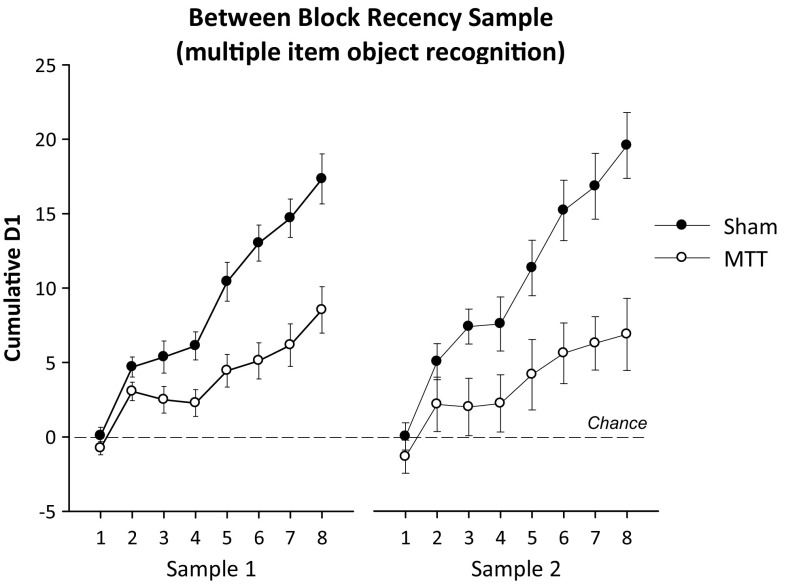
Cumulative D1 scores of the Sham and MTT groups across the sample phases of the multi-item between-block recency test during which the rats were tested on object recognition with a 1 min retention interval

The cumulative exploration time during the sample phases did not differ by lesion (*F* < 1). However, analysis of the cumulative D1 score (i.e., the cumulative difference in time spent exploring the novel versus the familiar objects during the sample phases) revealed a main effect of lesion (*F*_(1,21)_ = 12.6, *p* < 0.01) as well as a trial by lesion interaction (*F*_(7,147)_ = 16.5, *p* < 0.001), indicating that recognition performance involving continuous trials of standard object recognition with a 1-min delay was impaired in the MTTx relative to the Sham group (Fig. 4). This impairment was not absolute as performance in the MTTx group was above chance (i.e., 0) at the end of each sample phase (min *t*_(9)_ 2.8, *p* < 0.05).

**Fig. 5:**
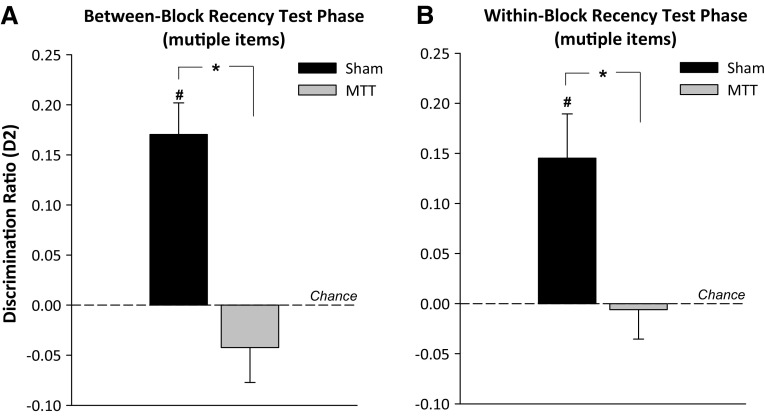
Updated D2 scores for the recency test phases of the between-block (**a**) and within-block (**b**) multi-item recency task. The mean D2 scores of the Sham and MTT groups are shown (±S.E.M.). *Hash* denotes significantly different from chance, *p* < 0.05. *Asterisks* denotes significant group difference, *p* < 0.05

In the test phase, Sham animals successfully discriminated between the objects on the basis of relative recency (i.e., a preferential exploration of objects from Sample 1 compared with Sample 2), whereas the MTTx group showed no preference for objects presented in Sample 1 (Fig. 5a). This difference was reflected by a main effect of lesion (*F*_(1,21)_ 20.5, *p* < 0.001). Test performance in Sham (*t*_(12)_ = 5.4, *p* < 0.001), but not MTTx (*t*_(9)_ = −1.2, *p* = 0.26) animals was above chance. However, overall exploration time during the test phase did not differ by lesion (*F* < 1).

##### Within-block recency

The cumulative exploration time during the sample phase did not differ by lesion group (*F* < 1). Similarly, there was no effect of lesion on overall levels of exploration during the test phase (*F* < 1).

Test performance was initially analysed by grouping the number of interleaving items into low (3 or 7 interleaving objects) or high (11 or 15 interleaving objects). ANOVA revealed no effect of the number of interleaving items on performance (*F* < 1) or an interaction with lesion group (*F* < 1). Consequently, the data were collapsed across the high and low interleaving items. As is clear from Fig. 5b, the MTTx group was impaired relative to the Sham animals (*F*_(1,21)_ = 7.1, *p* < 0.05). One-sample *t* tests confirmed that test performance in Sham animals was above chance (*t*_(12)_ = 3.3, *p* < 0.01), but performance in the MTTx group did not differ from chance (*t* < 0).

#### Experiment 1b: the effect of MTT lesions on single-item recency judgments

Total exploration time during both the sample and test phases did not differ by lesion group (*F* < 1).

**Fig. 6:**
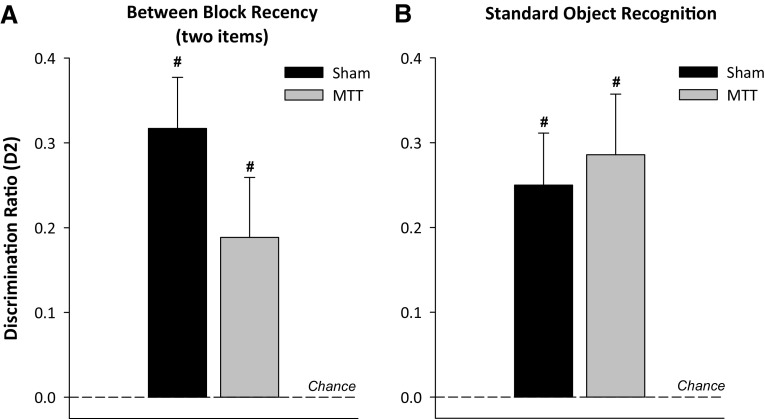
Mean D2 scores for the simple between-block recency test (**a**) and test of standard object recognition after a 60-min delay (**b**). The mean D2 scores of the Sham and MTT groups are shown (±S.E.M.). *Hash* denotes significantly different from chance, *p* < 0.05

In test phase, both groups showed a preference for the item that had been presented in the first temporal block (Fig. 6a) and there was no effect of lesion on test performance (*F*_(1,21)_ = 1.9, *p* = 0.2). One-sample *t* tests confirmed that both groups were able to make recency judgements about single items presented in distinct temporal blocks (Sham *t*_(12)_ = 5.3, *p* < 0.001; MTT *t*_(9)_ = 2.7, *p* < 0.05).

#### Experiment 1c: the effect of MTT lesions on standard object recognition

There was a non-significant trend towards overall higher levels of exploration during the sample phase in the MTTx group [(*F*_(1,21)_ = 3.6, *p* = 0.07; Mean total exploration time (±S.E.M.) Sham = 59.5 (±3.1); MTTx = 69.3 (±4.3)]. There was a similar trend during the test session [*F*_(1,21)_ = 3.9, *p* = 0.06; mean total exploration time (±S.E.M.) Sham = 36.6 (±2.2); MTTx = 44.4 (±3.5)].

Analysis of the D2 ratios from the test phase revealed that both Sham (*t*_(12)_ = 4.1 *p* < 0.01) and the MTTx animals (*t*_(9)_ = 4.1 *p* < 0.01) showed a preference for the novel object at test, indicating that both groups were able to discriminate objects in terms of relative familiarity after a 60-min delay (Fig. 6b). Object recognition performance did not differ by group (*F* < 1).

#### Experiment 2: the effect of crossed MTT-mPFC lesions on multi-item recency judgments

##### Between-block recency

There was no difference between the groups in the total cumulative exploration time in either sample phase (max *F*_(1,18)_ = 1.4, *p* = 0.24). Equally, total exploration time during the test phase was unaffected by lesion (*F* < 1).

**Fig. 7:**
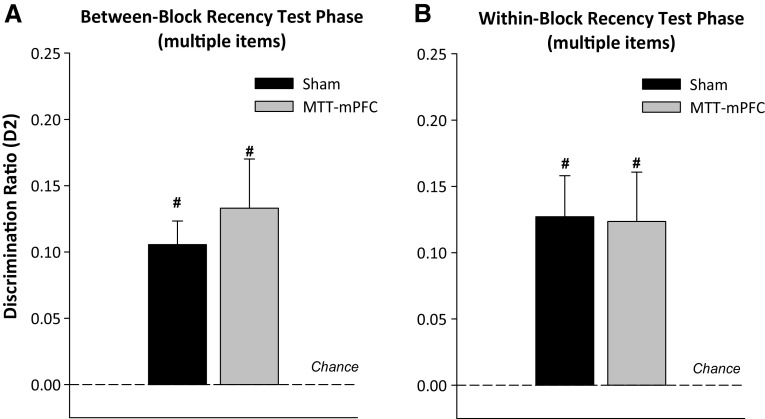
Updated D2 scores for the recency test phases of the between-block (**a**) and within-block (**b**) multi-item recency task. The mean D2 scores of the Sham and crossed MTT-mPFC groups are shown (±S.E.M.). *Hash* denotes significantly different from chance, *p* < 0.05

At test, both groups showed a preference for items that had been presented least recently (i.e., in Sample 1) (Fig. 7a). Performance in both Sham2 (*t*_(11)_ 5.9, *p* < 0.001) and the MTT-mPFC group (*t*_(7)_ = 4.4, *p* < 0.01) was above chance. There was no effect of lesion on test performance (*F* < 1).

##### Within-block recency

The total cumulative exploration time during the sample and test phases did not differ by lesion group (both *F* < 1).

The initial analysis revealed no effects of the number of interleaving items on test performance (*F* < 1), and consequently, the test data were collapsed across the number of interleaving items. As is clear from Fig. 7b, both Sham and MTT-mPFC groups were able to discriminate on the basis of relative recency between items presented within the same list and there was no difference between the groups (*F* < 1). One-sample *t* tests indicated that both groups showed a preference for the least recently presented (older) items (minimum *t*_(7)_ = 3.3, *p* < 0.05).

## Discussion

The mammillary bodies were first implicated in memory in the 19th century, but it is still unclear how they support memory (Kapur et al. 1996, 1998; Vann and Nelson 2015; Victor 1987). More broadly, there remains considerable controversy surrounding the core features of diencephalic amnesia as well as the critical site for amnesia to occur (Kopelman 2015). One consistent neuropsychological finding in diencephalic patients is impaired temporal/recency memory; but as the damage in this patient group is anatomically diffuse and often includes pathology beyond the medial diencephalon, a little progress has been made in establishing the precise neuropathology underpinning this deficit. This consideration underscores the importance of comparative lesion studies. Consequently, we examined the impact of selective transection of the rat mammillothalamic tract (MTT) on tests of recency memory analogous to the tasks used in patients (e.g., Hunkin et al. 2015). The distinct advantage of this approach is that it selectively disconnects mammillary body inputs to the anterior thalamic nuclei (Vann et al. 2007), while sparing other thalamic nuclei, such as the mediodorsal thalamus, intralaminar nuclei, and midline thalamic nuclei, that are often affected by the diffuse pathology seen in Korsakoff syndrome (Kopelman 2015; Mitchell and Chakraborty 2013). As such, this experimental approach provides a direct test of the selective importance of mammillary body efferents for recency memory. In line with the deficits observed in Korsakoff’s patients (Hunkin et al. 2015), the MTT lesion rats were severely impaired on tests of both ‘between’ and ‘within’ block recency memory. These results demonstrate for the first time the selective importance of the mammillary body efferents to the anterior thalamus for recency memory.

On both between- and within-block tests of recency memory, the MTT rats performed at chance level, consistently failing to discriminate between multiple objects on the basis of relative recency. An inability to recognise objects over a longer delay is an unlikely account of this pattern of results as the same animals performed at equivalent levels to sham animals on a test of object recognition after a 60-min delay (Fig. 6b). Similarly, when required to discriminate on the basis of relative recency between just two items that had been presented in different time blocks (Fig. 6a), performance in the MTT animals did not differ from sham levels. Even though these control tasks were run after the multiple-item recency tests, the lack of lesion effect is unlikely to reflect training related improvements given that the animals were at no point trained on a rule, i.e., they were not rewarded for choosing the correct (older) item so discrimination performance remained spontaneous throughout. Furthermore, there was no improvement when animals were moved from between-block to within-block testing. In contrast to the null effects on the simple object recognition task, a lesion impairment also emerged during the sample phases of the between-block task during which the animals were presented with multiple consecutive familiarity discriminations (Fig. 4), a procedure likely to increase stimulus interference. Interestingly, this deficit contrasts with the effect of hippocampal lesions which spare performance on tests of continuous object recognition (Albasser et al. 2012). Nevertheless, this deficit was not absolute as the MTT group’s performance remained above chance, i.e., these animals were still able to discriminate between multiple novel and familiar items, albeit less effectively than sham animals.

That impairments only emerged when the animals were required to make familiarity or recency judgements involving multiple objects indicates that impoverished recognition memory or a global deficit in processing temporal information cannot account for the effects of MTT damage on performance. Rather, this profile of deficits points to a specific problem with distinguishing between multiple items or events. One seemingly plausible explanation of this dissociation is that MTT damage leads to heightened sensitivity to the effects of proactive interference. Given that the most profound deficit emerged on the recency trials, it seems, however, unlikely that greater sensitivity to proactive interference can provide a complete account of this deficit, as this factor would presumably affect both familiarity and recency judgments alike. Similarly, the MTT animals were not differentially impaired on the within- relative to the between-block test, despite the latter test potentially reducing interference effects through the additional information provided by the distinct temporal blocks in which stimuli were presented. Moreover, evidence from delayed matching and non-matching to sample procedures provides a little support for the suggestion that mammillary body damage disrupts mnemonic processes through increased susceptibility to proactive interference (Aggleton et al. 1995; Harper et al. 1994; Vann and Aggleton 2003). Instead, the current findings indicate that the mammillary bodies are important for processing associative recognition information (Nelson and Vann 2014) and, in particular, the temporal context in which stimuli are presented. This suggestion accords with the clinical picture. While there have been a few reports of recognition memory deficits and abnormalities in the release from proactive interference in diencephalic amnesic patients (e.g., Kopelman and Stanhope 1998; Squire 1982), profound impairments in recency judgements have been reported in almost all Korsakoff cases (Kopelman 2015), and critically, these deficits have been observed in cases with intact familiarity judgements and normal sensitivity to proactive interference (Hildebrandt et al. 2001; Hunkin and Parkin 1993; Shaw and Aggleton 1995).

This impairment on recency tasks has typically been attributed to co-occurring frontal dysfunction (Mayes et al. 1985; Shimamura et al. 1990; Squire 1982) or disruption to thalamo-frontal circuits (Mair et al. 1979). While there is evidence that disrupting connections between the thalamus and frontal cortex can impair recency memory (Aggleton et al. 2011; Cross et al. 2012; Schnider et al. 1996), the current results demonstrate that restricted damage to the mammillary body-anterior thalamic axis that does not involve frontal associated thalamic nuclei, such as the mediodorsal thalamus, is in itself sufficient to produce marked impairments on tests of recent memory. However, damage to the medial diencephalon, in both rats and patients, can result in functional disruption to frontal cortex. There is very good evidence that diencephalic pathology can produce diaschisis in cortical regions, including frontal cortex (Baron et al. 1992; Fazio et al. 1992; Ozyurt et al. 2014; Paller et al. 1997; Pepin and Auray-Pepin 1993; Reed et al. 2003). Similarly, animal models have shown that selective MTT lesions can cause hypoactivity, as measured by immediate-early gene expression, in the prelimbic cortex (Vann and Albasser 2009; Vann 2013). The mammillary bodies can act indirectly on the prefrontal cortex via their connections with the anteromedial thalamic nucleus, which, in turn, projects to the prefrontal cortex (de Lima et al. 2016; Hoover and Vertes 2007; van Groen et al. 1999). Furthermore, the medial mammillary bodies are innervated directly by the medial prefrontal cortex (Allen and Hopkins 1989). It, therefore, remains possible that the present impairment seen following MTT lesions is driven by the loss of these direct and indirect prefrontal connections or by lesion-induced covert pathology and dysfunction in the prefrontal cortex. To examine the potential functional importance of interactions between the mammillary bodies and the medial prefrontal cortex for recency memory, we used disconnection procedures. In stark contrast to the effects of bilateral MTT lesions, the rats with MTT and contra-hemispherical medial prefrontal cortex lesions performed at normal levels on both the within- and between-block recency tasks. The implication is that the MTT lesion effects on tests of recency memory seen in Experiment 1 cannot be ascribed to either disconnection of the direct and indirect mammillary body-prefrontal pathway or MTT lesion-induced diaschisis. While there are undoubtedly differences between the human and rodent frontal cortex (Preuss 1995), there are, nevertheless, a number of functional consistencies across species (Uylings et al. 2003). Indeed, recency memory is sensitive to frontal damage in both rodents (e.g., Barker et al. 2007) and primates (e.g., Milner et al. 1991; Petrides 1991), making this an appropriate model to test these functional contributions. While the effects of prefrontal damage have not been tested on the multi-item tests of recency used in this study, it seems reasonable to assume that performance on these tasks would be sensitive to prefrontal damage given that prefrontal lesions consistently disrupt recency memory for single items presented in distinct temporal blocks (Barker et al. 2007; Cross et al. 2012; Hannesson et al. 2004; Nelson et al. 2011).

This study has addressed a long-standing and unresolved issue regarding the neuroanatomical basis of impoverished temporal memory in diencephalic amnesia. Using an animal model, we have shown that damage limited to the MTT can produce marked impairments on tests of recency memory analogous to those used in patients (Hunkin et al. 2015). Furthermore, the results of the disconnection study (Experiment 2) demonstrate that these effects are not due to the loss of interactions with the prefrontal cortex or distal effects of the MTT lesion on the prefrontal cortex. These results reveal for the first time the importance of the medial mammillary body inputs to the anterior thalamus for temporal discriminations. These findings point to the existence of two distinct but presumably complementary mechanisms for recency memory within the medial diencephalon: one involving the mammillary body-anterior thalamus axis (Dumont and Aggleton 2013; Wolff et al. 2006) and the other the mediodorsal thalamus (Aggleton et al. 2011; Cross et al. 2012; Mitchell and Chakraborty 2013; Mitchell and Dalrymple-Alford 2005). These distinct pathways can be dissociated behaviourally. Mediodorsal thalamic lesions, unlike damage to either the MTT or the anterior thalamus, disrupt recency memory for single items presented in distinct temporal blocks (Cross et al. 2012; Dumont and Aggleton 2013; Mitchell and Dalrymple-Alford 2005). Conversely, the role of the mammillary body-anterior thalamic axis would appear to be restricted to recency judgements involving multiple items (Dumont and Aggleton 2013). Furthermore, the mediodorsal thalamus and the prefrontal cortex appear to interact functionally to support simple between-block recency judgements (Cross et al. 2012). In contrast, the results from Experiment 2 suggest that the involvement of the mammillary bodies in recency memory does not require interactions with the prefrontal cortex. The presence of these distinct pathways may, in turn, explain why recency memory deficits are particularly prevalent in patient groups with medial diencephalic pathology. On the basis of the current results, it would seem likely that the mammillary bodies are required for fine-grained temporal discriminations when distinguishing between multiple stimuli. One potential role for the mammillary bodies in these processes may be through the regulation of theta, as the majority of cells in the medial mammillary nuclei modulate their firing rate at a frequency of theta (Bland et al. 1995; Dillingham et al. 2015a; Kocsis and Vertes 1994; Vann and Aggleton 2004). It has been suggested that theta provides an oscillatory activity pattern that may help separate temporal events (Dillingham et al. 2015b; Hasselmo and Eichenbaum 2005; Hasselmo and Stern 2014; Nyhus and Curran 2010). Mammillary body or MTT damage might, therefore, disrupt theta oscillations within Papez circuit, resulting in impaired temporal memory.
